# Editorial: Molecular mechanisms underlying pain relief and drug tolerance

**DOI:** 10.3389/fnmol.2023.1214264

**Published:** 2023-05-22

**Authors:** Quirin Krabichler, Ana Reynders

**Affiliations:** ^1^Department of Neuropeptide Research in Psychiatry, Central Institute of Mental Health, Medical Faculty Mannheim, University of Heidelberg, Mannheim, Germany; ^2^Aix-Marseille-Université, CNRS, Institut de Biologie du Développement de Marseille, Marseille, France

**Keywords:** pain, analgesia, planarians, morphine, oxytocin

Nociception is the process by which the nervous system detects and responds to noxious stimuli. Reaching brain and cortical areas, the nociceptive message might give rise to the sensation of pain. Noxious stimuli are detected by “nociceptors,” specialized primary afferent sensory neurons, which express various types of receptors and ion channels. Upon their activation, they release neurotransmitters which transmit pain signals to the spinal cord and the brain. There, these signals are processed and interpreted, leading to the perception of pain.

Pain is a highly averse sensation. It is present in different forms across the animal kingdom, although the quality of the sensation is thought to vary depending on the complexity of the animal's nervous system. Nevertheless, the basic neural and molecular mechanisms are homologous across the phylogenetic tree of animals. It is therefore both important and fruitful to study these mechanisms from the angle of comparative neurobiology—even more so, since much is still to be learned, for the sake of pure scientific curiosity, but especially for the sake of finding new cures. One of the major challenges in treating pain and especially chronic pain is that the use of established painkillers, such as opioids, non-steroidal anti-inflammatory drugs (NSAIDs), and local anesthetics, can lead to the development of tolerance, where the body becomes less responsive to the effects of the drug over time. This is thought to occur due to changes in the molecular mechanisms underlying nociception, such as increased expression of opioid receptors or desensitization of ion channels. As a result, higher doses of the drug are required to achieve the same level of pain relief, which can increase the risk of side effects and addiction.

New ways to treat pain will require improved understanding of the underlying mechanisms. Many molecular mechanisms of nociception have been known for decades, but amazingly, new ones continue to be discovered all the time. Many interesting novel findings relate to endogenous analgesic molecules released by various groups of neurons in the brain, which can inhibit pain transmission and provide pain relief. For example, Oxytocin (OT) has emerged as an intriguing new pain-killer molecule. Indeed, in addition to its well-established roles in mammalian reproduction and social interactions, this neuropeptide synthetized in the hypothalamus is increasingly acknowledged for its ability to regulate nociception at peripheral, spinal and supra-spinal sites. Accordingly, endogenous OT released following peripheral inflammation suppresses nociception and promotes analgesia. Moreover, exogenously administrated OT alleviates inflammatory and neuropathic pain. Apart from endogenous analgesics, many fascinating new discoveries are also still made with respect to classical and long-established painkillers such as opioids.

In this Frontiers in Molecular Neuroscience Research Topic “*Molecular mechanisms underlying pain relief and drug tolerance*,” five recent research articles are presented, which address a variety of current topics in pain research.

The study by Sun et al. focused on the analgesic actions of OT in the spinal cord. While the consensual view on the mechanisms underlying OT-mediated anti-nociception rely on the activation of oxytocin receptors (OTR) and the subsequent enhancement of inhibition of nociceptive inputs, this study provides a new mechanistic insight. Using a model of neuropathic pain, the authors propose that OT alleviates pain by promoting spinal GABA release and dampening presynaptic TRPV1-mediated neurotransmission. Electrophysiological recordings on acute spinal cord slices demonstrates that OT concomitantly increased the frequency and the amplitude of spontaneous GABAergic currents, while decreasing capsaicin-evoked EPSC. Importantly, this action was completely blocked by application of metabotropic GABA_B_ receptor antagonist, revealing an interaction of OT with spinal TRPV1-GABA_B_ system. Behavioral analysis of OT-injected naïve and neuropathic rats demonstrates the anti-nociceptive and analgesic properties of OT. Consistent with the electrophysiological results, OT normalized the up-regulation of TRPV1 induced by neuropathic lesion and had no effect when administrated to TRPV1 knock-out animals.

As further discussed by Gonzalez-Hernandez and Charlet in their commentary on the paper by Sun et al., these findings are consistent with another original study reporting a direct interaction of OT with TRPV1 *in vitro*, in heterologous cellular system and *in sillico*, in planar lipid bilayers. In this commentary, the authors summarize the knowledge on the mechanisms underlying OT-mediated anti-nociception and further underscore that the findings by Sun et al. illustrate a more complex than anticipated mode-of-action, in which several receptors, other than the classical OTR and various cellular targets, in addition to neurons may come into play.

These papers underscore the growing need to discover pain-relieving molecules. Indeed, one of the most widely used analgesic drug for the treatment of several pain conditions including inflammatory and neuropathic pain, is morphine. However, as illustrated by the recent opioid crisis in the United States, the use and efficiency of morphine is limited by side effects ranging from analgesic tolerance to life-threatening respiratory depression. Morphine tolerance arises following repeated drug administration and escalating doses are required to obtain the same analgesic effect. Despite advances in the elucidation of the mechanisms responsible for morphine tolerance, which include receptor internalization and de-sensitization, there are currently no therapeutic strategies to counteract this effect. In this context, Liu et al. examined the relevance of morphine-induced endoplasmic reticulum (ER) stress as a potential therapeutic target for morphine tolerance. They show that repetitive administration of morphine in rats induces tolerance, which in turns triggers the up-regulation of several ER stress sensor proteins and associated signaling pathways, including IRE1 and ATF6, in the spinal cord. Consistently, local inhibition of these pathways significantly attenuate morphine tolerance *in vivo*, providing a possible therapeutic basis.

Along the same line, Gabel et al., in their review of literature, discuss how morphine metabolism impacts on the efficiency of morphine analgesia. In particular, they focus on morphine-3-glucoronide (M3G), one of the main morphine-derived metabolites, which has been suggested to exhibit pro-nociceptive activity, thereby counter-acting morphine effect. Based on animal studies, the authors highlight several mechanisms likely to account for M3G pro-nociceptive action, including binding to morphine receptor and mobilization of the toll-like receptor 4 pathway. While these studies have a potential to increase the knowledge on the mechanisms underlying morphine side-effects, M3G effects in human patients appear less robust than those observed in rodents.

Finally, Reho et al. argue on the use of planarians as an original model system for the analysis of nociceptive behavior. Planarians are invertebrate “flatworms,” mostly studied in the field of regenerative medicine but also in drug abuse paradigms. More recent studies have provided evidence supporting the existence of a nociceptive system in planarians and of a subsequent nociceptive behavior. However, up-to-date, there is no consensus on how to experimentally measure such a behavior. In their review, the authors provide an exhaustive list of the known pharmacological compounds and their effect on planarians behavior. They further enumerate most of the terms used in dedicated studies, to describe planarians behavior. Overall, this review stands as a resource containing important information for the accurate use of planarians as a model system in the pain field.

## Author contributions

QK and AR wrote the editorial topic. All authors contributed to the article and approved the submitted version.

